# Parallelisms and Contrasts in the Diverse Ecologies of the *Anaplasma phagocytophilum* and *Borrelia burgdorferi* Complexes of Bacteria in the Far Western United States

**DOI:** 10.3390/vetsci3040026

**Published:** 2016-09-22

**Authors:** Nicole Stephenson, Janet Foley

**Affiliations:** School of Veterinary Medicine, Medicine and Epidemiology, University of California, Davis, CA 95616, USA; jefoley@ucdavis.edu

**Keywords:** *Anaplasma* spp., anaplasmosis, *Borrelia* spp., borreliosis, diversity, Lyme disease, reservoirs, western gray squirrel, woodrat

## Abstract

*Anaplasma phagocytophilum* and *Borrelia burgdorferi* are two tick-borne bacteria that cause disease in people and animals. For each of these bacteria, there is a complex of closely related genospecies and/or strains that are genetically distinct and have been shown through both observational and experimental studies to have different host tropisms. In this review we compare the known ecologies of these two bacterial complexes in the far western USA and find remarkable similarities, which will help us understand evolutionary histories and coadaptation among vertebrate host, tick vector, and bacteria. For both complexes, sensu stricto genospecies (those that infect humans) share a similar geographic range, are vectored mainly by ticks in the *Ixodes ricinus*-complex, utilize mainly white-footed mice (*Peromyscus leucopus*) as a reservoir in the eastern USA and tree squirrels in the far west, and tend to be generalists, infecting a wider variety of vertebrate host species. Other sensu lato genospecies within each complex are generally more specialized, occurring often in local enzootic cycles within a narrow range of vertebrate hosts and specialized vector species. We suggest that these similar ecologies may have arisen through utilization of a generalist tick species as a vector, resulting in a potentially more virulent generalist pathogen that spills over into humans, vs. utilization of a specialized tick vector on a particular vertebrate host species, promoting microbe specialization. Such tight host-vector-pathogen coupling could also facilitate high enzootic prevalence and the evolution of host immune-tolerance and bacterial avirulence.

## 1. Background

*Anaplasma phagocytophilum,* the causative agent of human granulocytic anaplasmosis, and *Borrelia burgdorferi,* the agent of Lyme disease, are obligately tick-transmitted bacterial zoonotic pathogens with remarkably similar ecologies [1]. Both occur throughout the Holarctic and are maintained and transmitted by ticks of the *Ixodes ricinus* subgroup: in each regional epidemiological cycle, the ticks (*I. pacificus* in the western USA, *I. scapularis* in the eastern and central USA, *I. ricinus* in Europe, and *I. persulcatus* in Asia) acquire the infection during their single larval or nymphal feeding on an infected small mammal, maintain the pathogen through the tick molt, and then transmit the pathogen to an uninfected host during the next stage as either nymph or adult. When infected nymphs feed on small mammals, they contribute to enzootic maintenance of the pathogen. There is minimal evidence that these pathogens can be transmitted transovarially among ticks [2,3,4].

However, beyond the apparent simplicity of a single regional bridge vector species is a highly complex web of numerous less well-studied, often host-specialist and nest-dwelling (nidicolous) ticks that vary in vector competence for these pathogens; a large diversity of mostly small mammal hosts variably capable of maintaining the bacteria and interacting with some but not all local tick vectors; and even a diverse set of bacterial strains and species with different (and usually poorly understood) host and vector specificity [5].

The name *B. burgdorferi sensu lato* (Bbsl) has been coined to refer to a clade of related genospecies, some causing disease consistent with Lyme disease, and others known from ticks and non-human vertebrates (Note, however, that the term genospecies varies in use, with some authors calling them genomospecies, some simply species, sometimes strains. We use strain to refer to an organism identified only tentatively, e.g., via a DNA sequence in a database, and use genospecies (some authors use “genomospecies”) to include any well-characterized pathogen in either the Bbsl or *A. phagocytophilum sensu lato* (Apsl) complexes). Included within Bbsl are the original agents attributed to Lyme disease, *B. burgdorferi sensu stricto* (Bbss), and *B. bissettii*, *B. californiensis*, *B. americana*, *B. carolinensis*, *B. kurtenbachii*, and *B. mayonii* among others. 

There are also distinct host-adapted *Anaplasma* strains but new species designations have rarely been erected, except for the recent description of *A. odocoilei* in deer [6]. Several distinct “strains” have been named AP-Variant 1 and DU1 [7,8]. Unfortunately, in prior studies, strains or species often were not correctly differentiated for a number of reasons [9,10]. Most importantly, the existence of distinct genospecies of these bacteria was not documented in the past and new genospecies continue to be discovered to the present day. Accurate insight into host-pathogen-vector relationships requires not only accurate differentiation of the genospecies but also extensive field and laboratory studies to examine the host and vector range and competence. In the absence of a catalogue of which bacterial genospecies occur in which hosts and ticks, attempts to synthesize studies, draw conclusions about reservoirs and maintenance or amplification cycles, and understand the global ecology and evolution of these bacterial complexes may be misguided. However, studies yielding insight into such differentiation have been performed in only a few systems, and those we include in this paper.

Highly biologically diverse systems offer a rich opportunity to examine the ecology of diseases where there are numerous intersecting enzootic cycles, which is the case in the western USA. California has 20 species of ticks in the *Ixodes* genus, including the known Bbsl vector-competent *I. pacificus* and *I. spinipalpis* and other relatively common small mammal-feeding species such as *I. woodi* and *I. angustus.* Small mammal diversity is high as well [7,11]. Our goals in this study were to compile all published data from California where genospecies or strain of *Anaplasma phagocytophilum* and *Borrelia burgdorferi* complexes have been differentiated in vertebrates or ticks. By systematically presenting the data, we aimed to review ecological features of each genospecies, focusing on the west but drawing comparisons where warranted to other regions and proposing likely epidemiologic cycles. Compiling and analyzing these data is intended to make reference to the diversity easier for future research projects and serve as a point from which future research can launch to further identify factors influencing the diversity of both of these different tick-borne pathogens to particular host and tick species.

## 2. Introduction to the *Anaplasma phagocytophilum* and *Borrelia burgdorferi* *sensu lato* Complexes

Delineating genetically and ecologically distinct genospecies and evaluating whether there are ecologically distinct host-vector-environment niches often lag far behind initial molecular detection and characterization. During early investigations of these tick-borne bacteria, numerous wildlife and domestic host and tick species were surveyed, but there was little available information on genetic diversity in the pathogens. In the case of *B. burgdorferi,* multiple closely related genospecies were assumed to be a single species. Alternatively, for *A. phagocytophilum* when the pathogen was found to be infecting multiple host species, it was not originally recognized to be a single agent (e.g., *E. equi*, *E. phagocytophila*, and the agent of HGE) [12]. As diagnostic and molecular techniques have progressed from serology (which often lacks specificity) to PCR, RFLP, and direct sequencing, to—most recently—MLST and full genome sequencing, we are able to more fully characterize and understand genetic diversity and postulate the evolutionary events that have led to our present-day pathogens. Reconciling published reports is thus challenging because older literature may fail to differentiate among distinct genospecies or use provisional isolate names (e.g., the agent of HGE or Genomospecies 1), which may later acquire valid scientific names. 

There are distinct host-adapted strains of *A. phagocytophilum* including *A. phagocytophilum sensu stricto* (Apss, also known as Ap-ha [8]), which is the strain known to cause disease in people, horses, dogs and cats [13] (Table 1). Other distinct strains and putative genospecies in the USA have been named DU1, AP-Variant 1, and WI-1, are associated with distinct wildlife species, and have not been reported in people. Experimental infection studies with Apss, DU1, and AP-Variant 1 have shown some restrictions on host specificity [8,14]. 

In contrast, the Bbsl complex has received more research attention and contains over 18 recognized or proposed genospecies and several more uncharacterized strains [15] (Table 2). Of those genospecies that are well supported, the main agents of human Lyme disease are Bbss in North America and BBss, *B. afzelii*, and *B. garinii* in Europe [16]. Other genospecies that have only been detected in sylvatic cycles among vertebrate hosts and tick vectors in California include *B. californiensis*, *B. americana*, and *B. carolinensis* [16]. Not yet characterized strains that fall outside of currently identified clades in California include Genomospecies 2 [17,18,19], R57-like, and CA690 [20].

## 3. Tick-Borne Sensu Stricto Strain/Genospecies Ecology

### 3.1. Reservoir Hosts

Due to obligate parasitism and the absence of transovarial transmission, vertebrate reservoir species are essential to the persistence of these tick-borne pathogens. In the eastern USA, the primary reservoir for both Apss and Bbss is the white-footed mouse (*Peromyscus leucopus*) [21,22,23,24,25], an r-selected species with a fast life-history pace that is abundant in many habitat types including those that have been disturbed. There are other competent hosts, such as the eastern chipmunk (*Tamias striatus*) for Apss [25], but because the primary reservoir is well suited for the pathogens and vectors, abundant, and broadly distributed geographically, the white-footed mouse per se is sufficient to maintain both pathogens. In California, the reservoir for both pathogens was originally thought to be the dusky-footed woodrat (*Neotoma fuscipes*) due to high infection prevalence [9,10,26]. As genetic testing became more advanced and strains were accurately identified, it became clear that the dusky-footed woodrat was in fact commonly infected with closely related strains that are not known to infect people, described below. Woodrats and *Peromyscus* spp. are relatively rarely infected with Bbss in most areas studied [27]. Rather, studies have shown a prevalence of Bbss in tree squirrels, primarily the native western gray squirrel (*Sciurus griseus*), as high as 50%–80% [20,27,28,29]. Reservoir competence studies have revealed that squirrels are sufficiently chronically infected that they maintain the pathogens in nature [29,30]. However, even though squirrels may have higher prevalence, less reservoir-competent species such as mice may be more abundant, amplifying their contribution to the overall infection risk.

The findings of one recent study stand out in contrast to earlier findings supporting western gray squirrels as reservoirs, although data are difficult to integrate because that study in two enzootic sites in Marin County did not include squirrels [31,32]. Of almost 700 woodrats and over 1000 deer mice (*P. maniculatus*) sampled (Table 2), Bbss prevalence was 13% and 6%, respectively, from which the authors suggested that these species might serve as reservoirs, even though much higher PCR prevalence (>50%) was typical in squirrels in sites where squirrels were tested, suggesting that if they had tested squirrels in Marin, a very high prevalence might have been found. These data reflect an important bias in much of the data available to date: woodrats and deer mice are much easier to trap and sample than western gray squirrels and so many more woodrats have been included in various studies than western gray squirrels [29,33]. 

Birds are thought to participate in local enzootic maintenance of Bbss as well as the transport of infected ticks [39,40]. In a recent study of over 600 birds and 50 species collected by mist netting in Mendocino County, Bbss was detected in the blood of 10 bird species, all in the order Passeriformes. Golden-crowned sparrows (*Zonotrichia atricapilla*) had the highest prevalence of Bbss (28.6%) and the authors suggested this species could be a reservoir [41]. Juvenile *I. pacificus* were collected from the birds in this study with a Bbss infection prevalence of 13% of 284 ticks. Ground-foraging behavior was most predictive of larval infestation, as has been shown previously [39,40,42]. 

As for the more commonly studied Bbss, the small mammals thought to be the reservoirs of Apss are tree squirrels and chipmunks [30,33]. Rejmanek et al. [7] first showed that the *A. phagocytophilum* strain infecting tree squirrels and chipmunks was most similar to that infecting humans, dogs, and horses and distinct from the strain infecting woodrats, known as DU1. Thus far, Apss has been detected in western gray squirrels, Douglas squirrels (*Tamiasciurus douglasii*), eastern gray squirrels (*S. carolinensis*), and the chipmunks *T. ochrogenys* and *T. sonomae* (Table 1) [7,34,35]. Apss has also been detected in 18 black bears (*Ursus americanus*) and a gray fox (*Urocyon cinereoargenteus*) in Humboldt County, but these are likely dead-end hosts if they experience short-duration infection and limited infestation by juvenile *I. pacificus* ticks [34,35,57]. The significance of incidental findings of Bbss-positive Virginia opossum (*Didelphis virginiana*) and black-tailed deer (*Odocoileus hemionus columbianus*) is unknown [28,48].

### 3.2. Clinical Disease

Symptoms of Apss infection in people, horses, dogs, and cats are typically mild to moderate, acute in onset, and self-limiting, with some cases likely going undiagnosed [24,58,59,60]. Clinical signs can include fever, lethargy, inappetance, and muscle pain. Apss can sometimes cause severe to fatal disease due to secondary complications such as acute respiratory distress syndrome, organ failure, sepsis, myocarditis, and hemorrhage (especially in immune-compromised individuals) [24,61,62]. The facts that infection with Apss may be relatively short-lived and that many clinical hosts experience bites from adult more frequently than nymphal ticks tend to reduce the likelihood that these hosts participate in pathogen maintenance cycles, although one study did show chronic infection in experimentally infected dogs [63].

In contrast to Apss, Bbss can cause more chronic disease in people with joint, cardiac, and neurologic complications, especially when treatment is not initiated early, though it is rarely fatal [64]. As with people, dogs can become persistently infected and suffer from prolonged clinical signs [65]. Additionally in dogs, there is a syndrome called Lyme nephritis, which is rare and fatal. The pathogenesis of this syndrome is poorly understood but thought to represent an immune-mediated reaction to the Borrelia organism which leads to kidney failure [66]. Lyme disease in horses and cats can be asymptomatic or be associated with a variety of clinical signs, which are similar to those seen in people and dogs including fever, lethargy, lameness, and neurologic signs [66,67].

### 3.3. Vectors

The main vector for both Apss and Bbss in the western USA is *I. pacificus*, a generalist tick known to feed on a wide variety of both small and large vertebrate hosts including domestic animals and people, making it an ideal bridge vector [68,69]. Like all *I. ricinus*-complex ticks, *I. pacificus* has three host-feeding stages. It feeds primarily on small mammals and lizards during its immature stages and larger mammal species as an adult [57]. It tends to be a host-generalist tick, willing to feed on any of the dozens of species it encounters. It quests on the ground or in open vegetation, ascending grass stems or bushes daily during questing season to seek hosts. It takes refuge in leaf litter if a host is not found or, once fed, while waiting through a molt or laying eggs. 

In California, Apss has exclusively been detected in *I. pacificus,* although only two studies have attempted to differentiate genospecies among ticks in the western USA (Table 1) [13,36]. Documented prevalences of Bbss in *I. pacificus* in California generally range from <1% to 5%, although prevalences as high as 8%–10% have been found in some “hotspot” areas in Marin, Sonoma, and Mendocino Counties [31,44,46,47]. There have been rare descriptions of Bbss in *I. auritulus*, *I. spinipalpis*, *I. jellisoni*, and *Dermacentor occidentalis* (Table 2), though the latter tick has been shown to be incapable of transmitting the pathogen either transovarially or to a vertebrate host [70]. Bbss has been detected in an *I. angustus* in the northwestern USA and British Columbia [71,72,73], and this tick species was removed from the eyelid of a three-year-old girl in Washington State who developed an erythematous rash 23 days later and six months later had an elevated serum titer to *B. burgdorferi* by IFA [74]. *I. angustus* has been experimentally shown to be vector-competent for Bbss using deer mice as a host model [75] and does occasionally feed on people [57], so it may play a small role as a bridge vector, likely where other more competent vectors are less common. 

In much of California, the western gray squirrel and lizards appear to be the preferred hosts of juvenile *I. pacificus*. One study showed that western fence lizards (*Sceloporus occidentalis*) and southern alligator lizards (*Elgaria multicarinata*) may host up to 90% of juvenile *I. pacificus* (Casher 2002). Despite increasing the carrying capacity for *I. pacificus*, lizards may reduce the force of infection of Bbss and possibly Apss in California by a zooprophylactic effect, by diverting questing juvenile *I. pacificus* from reservoir competent hosts and failing to succumb to infection. This is evidenced by PCR surveys of lizards failing to show any significant Apss infection [76]. In addition, experimental studies showed that western fence lizards did not become infected with Apss [76] and their blood was borreliacidal, clearing *Borrelia* infections in the ticks that were feeding on them [77]. Through this mechanism, lizards may ultimately decrease the density of *infected* nymphs and therefore risk of disease to people, even though one study’s results did not support this hypothesis [78]. This contrasts, however, with Bbsl in Europe, where *B. lusitaniae* is reservoired by lizards [79].

## 4. Ecology of Other Sensu Lato Strains/Genospecies

### 4.1. Anaplasma Phagocytophilum Sensu Lato 

In North America, three strains that are distinctly different from Apss have been documented: strain DU1 in small mammals, WI-1 in deer, and the AP-Variant 1 also in deer. The Apsl strain DU1 was originally detected in a woodrat from Mendocino County in coastal northern California [7]. It is found in woodrats throughout northern and central California and is genetically distinct from Apss in 23S-5S intergenic spacer, *ank*, and *groESL* markers. DU1 has occasionally been detected in black bears, redwood chipmunks, and western gray squirrels (Table 1) [7,13,34,35]. It has also been detected in an *I. ochotonae* that was attached to a deer mouse, an *I. woodi* and an *I. angustus* that were attached to woodrats, and two *I. pacificus*, one that was attached to a redwood chipmunk and one that was found on a person [13]. Experimental infection studies confirmed that DU1 did not infect horses, a proxy for clinical hosts including humans [14]. A plausible theory is that this pathogen circulates in primary enzootic cycles involving the dusky-footed woodrat and the nidicolous tick *I. spinipalpis* with spillover into chipmunks and other tick species, although, thus far, few studies have been performed that provide sequence data or have looked specifically for this strain.

First detected in white-tailed deer (*Odocoileus virginianus*) in Wisconsin, DNA of the WI-1 strain of Apsl was found in *D. albipictu*s in Minnesota, which appeared to be capable of transovarial transmission of the bacterium [80]. A genetically similar or identical strain was also detected in black-tailed deer and keds (*Lipoptena depressa*) in Mendocino, Mono, and Tehama Counties in California [36]. *D. albipictus* is a specialist tick on cervids and may be the primary vector for this Apsl strain, although it is not a tick that feeds on humans and is infrequently collected and tested for human pathogens. Vector competence for Apsl has not been assessed in keds, which are flies that lose their wings once they alight on a deer, whose blood they feed on and become obligate parasites [81].

**Figure 1:**
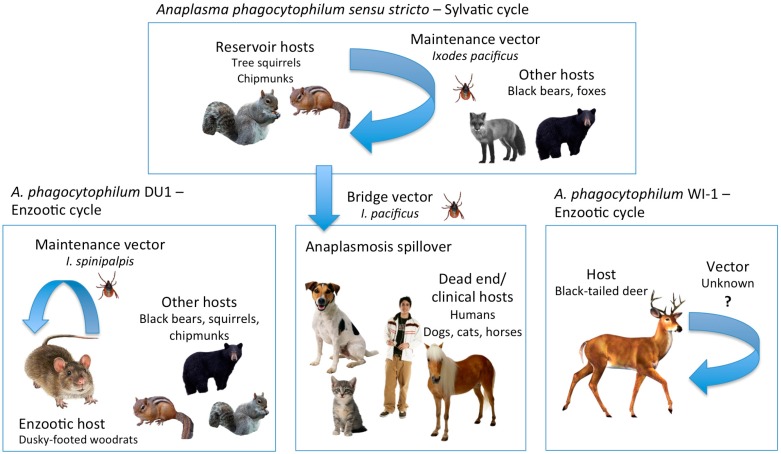
Proposed epidemiological cycles of the *Anaplasma phagocytophilum* *sensu lato* complex in California.

A well-characterized strain of Apsl that also appears to have an ungulate-tropism, designated AP-Variant 1, has not been found to date in California. This strain has been found in the eastern USA in white-tailed deer and *I. scapularis* ticks [8]. Experiments confirmed that it is unable to infect rodents but did infect goats; it was inferred that AP-Variant 1 is unlikely to be able to infect humans [82,83]. An interesting question is whether this strain could infect cattle. The strain was reported as present in a black-tailed deer in Mendocino County [37], but re-examination of the DNA sequence from that case with the more comprehensive database available now in GenBank indicated that the deer was actually infected with the strain WI-1 (Stephenson, unpub. data). It is not known whether *I. pacificus* is an incompetent vector, the strain is not present in California, or whether it is uncommon and not yet discovered. Other *Anaplasma* genospecies that have been detected in the western USA after being initially mislabeled as *A. phagocytophilum* include *A. ovis*, *A. bovis,* and *A. odocoilei* [36,37]. Figure 1 depicts the proposed epidemiologic cycles of the *A. phagocytophilum sensu lato* complex in California based on the currently available compiled data.

### 4.2. Borrelia Burgdorferi Sensu Lato

**Figure 2:**
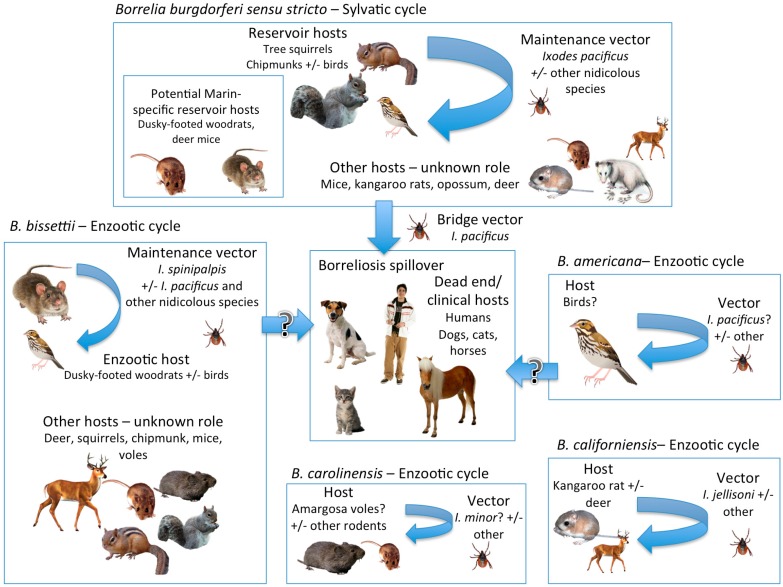
Proposed epidemiological cycles of the *Borrelia burgdorferi* *sensu lato* complex in California.

Figure 2 depicts the proposed epidemiologic cycles of the *B. burgdorferi sensu lato* complex in California based on the currently available compiled data. *B. carolinensis* is a Bbsl genospecies not known to affect humans, mainly found in the southeastern USA, where it has been detected in cotton mice (*P. gossypinus*), eastern woodrats (*N. floridana*), and a single *I. minor* [84]. *I. minor* is abundant in the southeastern USA and feeds primarily on small rodents and ground-feeding birds [85]. The nomenclature of this tick has been controversial but its first, poorly substantiated record was from Guatemala and it is listed as part of the tick fauna in Panama [86,87]. Recently, Foley et al. [56] described an enzootic cycle of *B. carolinensis* in the Mojave Desert, California, involving *I. minor* and the endangered Amargosa vole (*Microtus californicus scirpensis*), which are geographically isolated in small marshes in the desert. Further evaluation of this tick indicates that it is subtly morphologically distinct from southeastern *I. minor*, and may constitute a not previously identified species [88]. A likely route for its introduction into the Mojave Desert is via migratory birds. 

*Borrelia americana* was first isolated from an *I. pacificus* collected from California in 1993, although at that time it was described as “genomospecies 1” [89]. Since then it has been detected mainly in *I. minor*, the eastern towhee (*Pipilo erythrophthalmus*), and the Carolina wren (*Thryothorus ludovicianus*) from South Carolina [89]. Passerine migration may account for the spread not only of *I. minor* but also *B. americana.* In fact, in 2016 Scott and Foley [90] reported the first finding of *B. americana* in Canada in an *I. auritulus* from a resident bird in Ontario, further suggesting expansion of this *Borrelia* genospecies into the New World. An alternative explanation may be that *B. americana* has been present in largely overlooked ecologies and is only now being detected. *I. auritulus* typically infests birds, while *I. minor* is a tick of birds and small mammals, neither of which typically bite humans. However, spillover into an ecology featuring *I. pacificus* could put humans and other clinically affected hosts at risk. At least one study has detected *B. americana* in the blood of two ill patients, but the role that this species plays in disease is still unclear [91]. 

As of 2016, *B. californiensis* has only been detected in Mendocino County in coastal northern California, where it mainly infects the California kangaroo rat (*Dipodomys californicus*) and its specialist tick *I. jellisoni* [89]. It was also detected in two black-tailed deer, an *I. pacificus* and *I. spinipalpis* that were attached to kangaroo rats, and a flagged *I. pacificus* [15,43,48]. *B. californiensis* has not been associated with human cases of Lyme disease [15].

Unlike most genospecies of Bbsl, *B. bissettii* has characteristics similar to Bbss in that it has a broader vertebrate host range, utilizes generalist vectors in the *I. ricinus* complex, and is found on multiple continents (both North America and Europe). *B. bissettii* has been implicated in several cases of Lyme disease in eastern Europe with *B. bissettii* DNA detected in human patients with symptoms typical of Lyme disease including erythematous rash, fatigue, joint pain, and both cardiac and neurologic abnormalities [92,93,94] and in the cerebrospinal fluid of a German patient with no history of travel [95]. In California, *B. bissettii* has been detected in the sera of residents of Mendocino County, an area with high prevalence of Bbss, but was not associated with clinical illness [96]. 

*Borrelia bissettii* circulates within at least two restricted, local enzootic host-vector cycles [15]. Although the *B. bissettii* type strain DN127 was first isolated from an *I. pacificus*, subsequent research showed that it was maintained enzootically in nidicolous *I. spinipalpis* and woodrats in California and Colorado [43]. This same tick-host couplet maintains the Apsl strain DU1 in California and an un-genotyped *A. phagocytophilum* strain in an area of Colorado where neither *I. pacificus* nor *I. scapularis* bridge vectors are present [97]. As for Bbss, *B. bissettii* was isolated from several bird species as well as the juvenile stages of *I. pacificus* that were attached to birds [41]. However, the prevalence of *B. bissettii* in ticks from birds was much lower than Bbss, and the role of birds as a possible reservoir for *B. bissettii* is not resolved. *B. bissettii* has most commonly been detected in the tick *I. pacificus*, though this may be due to biased sampling as *I. pacificus* is the most commonly flagged *Ixodes* sp. tick in California. *B. bissettii* has also been detected several times in *I. spinipalpis* that were collected both by flagging and attached to vertebrate hosts as well as once each from an *I. jellisoni* and *I. auritulus* (Table 2). Altogether, the data suggest that *I. spinipalpis* may be the main vector responsible for enzootic maintenance and *I. pacificus* may contribute to enzootic transmission and as a potential bridge vector to people.

The best characterized but not yet named Bbsl strain in California is “Genomospecies 2”. Genomospecies 2 has been detected by PCR and sequencing only in California, initially in an *I. spinipalpis* tick collected from a black-tailed jackrabbit (*Lepus californicus*) in Mendocino County sometime between 1984 and 1989 [89], later (1993) detected in an *I. pacificus* individual flagged in Kern County, likely in the Sierra Nevada foothills [17], and most recently (between 2009 and 2012) in an *I. pacificus* flagged in Alameda County [20]. This is a broad geographical distribution that encompasses relatively moist low-altitude coastal mountains, across the dry Central Valley, which is generally inhospitable to many *Ixodes* spp. ticks, to the Sierra Nevada foothills hundreds of kilometers to the southeast of the coast. Genomospecies 2 has thus far not been detected in any vertebrate species. 

## 5. Discussion

In California, with its rich diversity in habitat types, plants, animals, and other biological taxa, it is not surprising that there should be high diversity in tick-borne bacteria and in ecological transmission cycles. Here we review the available data where pathogen genospecies have been identified in California and find multiple important generalities. For both pathogens, sensu stricto genospecies tend to have a broader host range including people, utilizing mainly tree squirrels and chipmunks as reservoirs compared with other sensu lato genospecies. Both Apss and Bbss have a broad geographic range occurring in both the Old and New Worlds, utilizing white-footed mice as reservoirs in the eastern and midwestern USA. In California, the primary “bridge vector” for both Bbss and Apss is *I. pacificus,* although sensu stricto genospecies are occasionally detected in a variety of other *Ixodes* spp. ticks. In contrast, most other sensu lato genospecies tend to have narrower host ranges but are found in a larger diversity of *Ixodes* spp. ticks, including *I. pacificus*, though at much lower prevalence than the sensu stricto strain. Other sensu lato strains often occur in local or more geographically restricted areas. 

In the west, both Bbsl and Apsl complexes appear to have local enzootic cycles between woodrats and the nidicolous tick *I. spinipalpis*. This probably reflects the tight ecological relationship of woodrat ticks (*I. spinipalpis* and *I. woodi,* although tick-borne pathogens have been detected more in *I. spinipalpis*), which in many areas have a nidicolous habit: when not on the animal, they reside primarily in nest cups within woodrat stick or underground houses. Especially for the territorial dusky-footed woodrat, each house is defended by a single woodrat individual and whether or not all houses in an area are occupied varies seasonally and annually [98]. Nest occupation improves tick survival but also limits access to hosts, although various *Peromyscus* spp. will sometimes invade woodrat houses and become infested [98]. Within such a relationship, pathogens may become specialized to their hosts and this may manifest in the evolution of avirulence by the pathogen or the woodrat immune system becoming more tolerant, allowing for chronic infection. This may extend the vector’s window of transmission and increase the prevalence (and success) of the pathogen in the population. An example of this phenomenon is that the *OspC* genes of *B. afzelii* and *B. garinii* appear to be optimized for their respective typical hosts, only inducing strong immunity in the host typical of the opposite pathogen, and resulting in virtually no intermediate genotypes when the two pathogens coinfect [99]. We have also noted that woodrats show remarkably high PCR prevalence in some areas without being seropositive, a possible indicator of failing to mount strong immune responses to infection likely because woodrat-tick-borne bacterial coevolution has now resulted in these bacteria functioning more as commensals than parasites for this host species [100]. 

California has a higher diversity of Sciuridae—tree squirrels and chipmunks—than any place in the world. The five tree squirrel species in California include the widely distributed and large-bodied western gray squirrel, its two non-native but similar competitors, the eastern gray and fox squirrels (*S. carolinensis* and *S. niger*), and the smaller Douglas and northern flying squirrels, which occur in deep forests in the northern and eastern parts of the state. The relatively long-lived western gray squirrel is a reservoir of both Apss and Bbss in part because it is an important host for the juvenile stages of the cosmopolitan *I. pacificus* [57]. Notably, while *I. pacificus* is a broadly distributed and locally abundant vector-competent tick that preferentially feeds on reservoir-incompetent reptiles in larval and nymphal stages, some immature individuals feed on small mammals or birds but may be outcompeted by more specialized tick-species in some cases [68]. The sciurid specialist tick species *I. hearlei* is rarely encountered in California, potentially leaving an open niche that *I. pacificus* has filled [57,101]. In sites where comparative tick infestation has been assessed, the *I. pacificus* load is more than four times higher on squirrels than in woodrats and almost 15 times higher than *Peromyscus* spp. [40]. Tree squirrels could encounter *I. pacificus* on the ground or tree trunks, which have proven to have high loads of questing nymphal ticks [102]. In light of the high numbers of Apss and Bbss in western gray squirrels in many areas, where infections in woodrats and deer mice may be an order of magnitude lower or undetectable, the most probable explanation for the finding in Swei et al. 2011, that woodrats and deer mice could be “reservoirs” is that infection rates in squirrels would likely have been even higher had they been trapped and tested [31,32]. 

While western gray squirrels have been clearly shown to be reservoir-competent for Bbss using experimental infections [29], this has not been done for Apss in part because of the considerable difficulty in capturing and keeping this species in captivity. Doing such a study would offer valuable opportunities to assess immune impacts of both tick infestation and pathogen infection in this species as well. In contrast, however, reservoir competence for Apss has been documented in one of the species of chipmunk known to have very high infection prevalence, the redwood chipmunk [30], as well as the eastern gray squirrel [25]. Interestingly, although *I. angustus* is abundant on Allen’s (*T. senex*), redwood, and Siskiyou (*T. siskiyou*) chipmunks in northern coastal sites where Apss is found, this tick is quite rarely found infected with the pathogen [103]. Nevertheless, it is known to occasionally bite humans, has also been shown to carry Bbss, and was implicated in a human case of Lyme disease [74].

Despite the many similarities between the ecologies of these two tick-borne pathogens, there are also differences. Borreliosis tends to cause chronic infections in both humans and animals, especially when untreated, compared to anaplasmosis. For enzootic cycles this likely means that reservoir species, once infected, are infectious to vectors for the rest of their life. In contrast, Apss is generally acute and self-limiting in most clinical hosts and often transient in reservoirs [26,30], although one study in dogs did document persistent infection until the end of the study (60 days) without the administration of antibiotics [63]. Very limited data are available on the relative persistence of infection among various Apsl genospecies.

Another difference is that enzootic cycles within the Bbsl complex tended to associate with rodent and sometimes bird species, with only Bbss, *B. bissettii*, and *B. californiensis* being detected in deer [48]. In the Apsl complex, although DU1 is a rodent-adapted strain, WI-1 and Ap-Variant 1 are both ungulate-adapted, as are the majority of strains in Europe [104]. In addition, *A. ovis*, *A. odocoilei*, and *A. marginale*, all closely related species, are ungulate-adapted as well [6]. The main vector of Bbsl to birds is likely *I. pacificus,* and in fact the main genospecies in birds is typically Bbss, also reported from *I. auritulus* and *I. spinipalpis*, which bite birds as well [57]. However, these same ground-foraging birds are exposed to ticks carrying Apss but there is limited evidence suggesting birds as reservoirs for Apsl [42]. Further studies are needed to account for these differences, but a preliminary hypothesis could rely on original ancestral genospecies, if the *Anaplasma* genus was originally an ungulate pathogen, while *Borrelia* may have expanded from early relationships with rodents. 

Important limitations in the data must be addressed in order to continue to understand their ecologies. Now that it is clear that there are distinct genospecies of both pathogens, older studies where the genospecies was not identified may be difficult to interpret. The studies included in this review employed different methodology to study these pathogens, making it hard to synthesize the data. *I. pacificus* is largely overrepresented in the data in part because, as the vector mainly responsible for transmission to people, it is of highest public health interest. Additionally, this is the most likely *Ixodes* spp. to collect by flagging. Other species of ticks are generally collected from trapped vertebrate hosts, which requires greater effort than flagging and is biased by the vertebrate species being targeted. Some small mammal species, such as woodrats and deer mice, are very easy to trap, which leads to their over-representation in the data, especially when compared to tree squirrels. For some hosts, vectors, and bacterial genospecies, competence studies have not been performed. There is a strong emphasis on data from particular geographical locations, either because active researchers focused their attention on particular sites or these sites appeared to be “hotspots” for infection, for example Hopland Field Station and Hendy Woods State Park in Mendocino County. Valuable data could be compiled if larger, systematic surveys were conducted of more species, over wider and possibly randomly selected areas, and if data were reported not mostly as case reports but more as systematic prevalence estimates. 

A rich microbial flora embedded within rich tick and host fauna impacts prospects for pathogens to enhance or reduce other pathogens’ forces of infection and the likelihood that coinfection might change clinical impacts on hosts. In multiple regions, coinfection of Bbsl and Apsl in ticks is more common than expected by chance [105]. However, the order in which exposure to different pathogens or genospecies occurs may impact the outcome. Genospecies that use nidicolous hosts are more likely to be the first that their specialist hosts encounter and, while these hosts may tolerate coadapted genospecies, they might respond differently when subsequently challenged with a new genospecies such as a sensu stricto genospecies in a generalist tick. Using nidicolous ticks may lead to a higher prevalence of the coadapted genospecies in a host species, at the expense of the generalist genospecies. The availability of multiple nidicolous tick-specialist host dyads in a biodiverse system ensures polymorphic niches that genospecies can occupy and helps support the biodiversity in bacterial pathogens. A similar pattern is seen in England, where a deer—*I. ricinus* cycle maintains a genetically distinct subpopulation of *A. phagocytophilum*, which overlaps a separate cycle involving field voles (*Microtus agrestis*), *I. trianguliceps*, and a variant pathogen strain [106,107], as well as in rodent and insectivore cycles in Europe and Asia [108,109,110]. In contrast, the fact that both *B. burgdorferi* and *A. phagocytophilum* in the eastern USA are primarily transmitted by one of the most common and most cosmopolitan ticks (*I. scapularis*) and reservoired by a widespread rodent with notably fast life history may serve to diminish pathogen diversity. 

Systematically distinguishing ecologically and epidemiologically relevant genospecies is essential in future research and disease surveillance and control. A better understanding of what underlying ecological and evolutionary drivers influence such diverse pathogen persistence will allow public health workers and biologists to better predict different disease patterns in time and space.

## Figures and Tables

**Table 1:** List of strains within the *Anaplasma phagocytophilum sensu lato* complex in California, their distribution, host and vector range, and number of times each has been documented.

Strain	Counties	Species (Host or Vector/Host) ^1^	Number of Occurrences ^2^	References
*Sensu stricto*	Alameda, El Dorado, Humboldt, Placer, Napa, Marin, Mendocino, Santa Cruz, Shasta, Yolo	*Ursus americanus*	18	[7,13,34,35]
		*Equus caballus*	10	
		*Canis lupus familiaris*	6	
		*Sciurus griseus*	4	
		*Tamias sonomae*	2	
		*T. ochrogenys*	2	
		*Tamiasciurus douglasii*	2	
		*S. carolinensis*	2	
		*Urocyon cinereoargenteus*	1	
		*Ixodes pacificus/flag*	4	
		*I. pacificus/Neotoma fuscipes*	1	
DU1	Humboldt, Mendocino, Santa Cruz	*N. fuscipes*	28	[7,13,34,35]
		*U. americanus*	8	
		*T. ochrogenys*	4	
		*S. griseus*	1	
		*I. spinipalpis/N. fuscipes*	3	
		*I. angustus/N. fuscipes*	1	
		*I. woodi/N. fuscipes*	1	
		*I. ochotonae/Peromyscus* sp*.*	1	
		*I. pacificus/T. ochrogenys*	1	
		*I. pacificus/Homo sapiens*	1	
WI-1	Mendocino, Mono, Tehama	*Odocoileus hemionus*	16	[36,37,38]
		*Lipoptena depressa/O. hemionus*	10	

**^1^** For tick species that were collected either by flag or from a vertebrate host, this is denoted after the tick species name; **^2^** Number of occurrences refers to the number of molecular detections of the strain in tissue from an individual host or vector.

**Table 2:** List of genospecies within the *Borrelia burgdorferi sensu lato* complex in California, their distribution, host and vector range, and number of times each has been documented.

Genospecies	Counties	Species (Host or Vector/Host) ^1^	Number of Occurrences ^2^	References
*B. burgdorferi*	Alameda, Butte, Contra Costa, El Dorado, Humboldt, Lake, Los Angeles, Marin, Mendocino, Napa, Placer, Plumas, Sacramento, San Mateo, Santa Clara, Santa Cruz, Shasta, Sonoma, Tehama, Trinity	*Neotoma fuscipes*	95	[17,20,27,28,29,41,43,44,45,46,47,48,49,50] ^3^
		*Peromyscus maniculatus*	59	
		*Sciurus griseus*	38	
		*S. niger*	19	
		Birds	14	
		*Tamias senex*	4	
		*Tamiasciurus douglasii*	2	
		*T. ochrogenys*	2	
		*P. boylii*	2	
		*P. trueii*	2	
		*Dipodomys californicus*	1	
		*S. carolinensis*	1	
		*Didelphis virginiana*	1	
		*Odocoileus hemionus*	1	
		*Ixodes pacificus*/flag	1095	
		*I. pacificus/S. griseus*	25	
		*I. pacificus/birds*	3	
		*Dermacentor occidentalis*/flag	2	
		*I. auritulus*/flag	1	
		*I. spinipalpis*/flag	1	
		*I. jellisoni*/flag	1	
*B. bissettii*	Alameda, Contra Costa, Del Norte, Humboldt, Mendocino, San Bernardino, San Luis Obispo	*N. fuscipes*	72	[15,20,27,29,41,43,48,49,51,52,53,54] ^2^
		Birds	17	
		*P. boylii*	10	
		*O. hemionus*	8	
		*P. trueii*	7	
		*P. maniculatus*	5	
		*T. siskiyou*	4	
		*N. lepida*	3	
		*R. rattus*	3	
		*M. californicus*	2	
		*T. douglasii*	1	
		*I. pacificus*/flag	49	
		*I. spinipalpis*/flag	12	
		*I. pacificus*/bird	5	
		*I. pacificus/S. griseus*	3	
		*I. spinipalpis/N. fuscipes*	2	
		*I. jellisoni*/flag	1	
		*I. auritulus*/flag	1	
*B. californiensis*	Alameda, Mendocino	*D. californicus*	19	[15,20,48]
		*O. hemionus*	3	
		*I. jellisoni/D. californicus*	1	
		*I. spinipalpis/D. californicus*	1	
		*I. pacificus/D. californicus*	1	
		*I. pacificus*/flag	1	
*B. americana*	Alameda, El Dorado, Los Angeles, Orange	*I. pacificus*/flag	4	[17,20,55]
	
*B. carolinensis*	Inyo	*I. minor/Microtus californicus*	1	[56]

**^1^** For tick species that were collected either by flag or from a vertebrate host, this is deno*ted after the tick species name*; **^2^** Number of occurrences refers to the number of molecular detections of the genospecies in tissue from an individual host or vector; **^3^** Supplemented with unpublished data from Foley and Roy, UC Davis.

## References

[1] Brown R.N., Lane R.S., Dennis D. (2005). Geographic Distributions of Tick-Borne Diseases and Their Vectors.

[2] Foley J.E., Foley P., Brown R.N., Lane R.S., Dumler J.S., Madigan J.E. (2004). Ecology of granulocytic ehrlichiosis and Lyme disease in the western United States. J. Vector Ecol..

[3] Munderloh U., Kurtti T. (1995). Cellular and molecular interrelationships between ticks and prokaryotic tick-borne pathogens. Ann. Rev. Entomol..

[4] Rollend L., Fish D., Childs J.E. (2013). Transovarial transmission of *Borrelia* spirochetes by *Ixodes scapularis*: A summary of the literature and recent observations. Ticks Tick-Borne Dis..

[5] Foley J., Rejmanek D., Fleer K., Nieto N. (2011). Nidicolous ticks of small mammals in *Anaplasma phagocytophilum*-enzootic sites in northern California. Ticks Tick-Borne Dis..

[6] Tate C.M., Howerth E.W., Mead D.G., Dugan V.G., Luttrell M.P., Sahora A.I., Munderloh U.G., Davidson W.R., Yabsley M.J. (2013). *Anaplasma odocoilei* sp. nov. (family Anaplasmataceae) from white-tailed deer *Odocoileus virginianus*. Ticks Tick-Borne Dis..

[7] Rejmanek D., Bradburd G., Foley J.E. (2012). Molecular characterization reveals distinct genospecies of *Anaplasma phagocytophilum* from diverse north American hosts. J. Med. Microbiol..

[8] Massung R.F., Priestley R.A., Miller N.J., Mather T.N., Levin M.L. (2003). Inability of a variant strain of *Anaplasma phagocytophilum* to infect mice. J. Infect. Dis..

[9] Brown R.N., Lane R.S. (1992). Lyme disease in California: A novel enzootic transmission cycle of *Borrelia burgdorferi*. Science.

[10] Nicholson W.L., Castro M.B., Kramer V.L., Sumner J.W., Childs J.E. (1999). Dusky-footed wood rats (*Neotoma fuscipes*) as reservoirs of granulocytic Ehrlichiae (Rickettsiales: Ehrlichieae) in northern California. J. Clin. Microbiol..

[11] Foley J.E., Nieto N.C., Massung R., Barbet A., Madigan J., Brown R.N. (2009). Distinct ecologically relevant strains of *Anaplasma phagocytophilum*. Emerg. Infect. Dis..

[12] Dumler J.S., Barbet A.F., Bekker C.P.J., Dasch G.A., Palmer G.H., Ray S.C., Rikihisa Y., Rurangirwa F.R. (2001). Reorganization of genera in the families Rickettsiaceae and Anaplasmataceae in the order Rickettsiales: Unification of some species of *Ehrlichia* with *Anaplasma*, *Cowdria* with *Ehrlichia* and *Ehrlichia* with *Neorickettsia*, descriptions of six new species combinations and designation of *Ehrlichia equi* and “HGE agent” as subjective synonyms of *Ehrlichia phagocytophila*. Int. J. Syst. Evol. Microbiol..

[13] Rejmanek D., Freycon P., Bradburd G., Dinstell J., Foley J. (2013). Unique strains of *Anaplasma phagocytophilum* segregate among diverse questing and non-questing *Ixodes* tick species in the western United States. Ticks Tick-Borne Dis..

[14] Foley J., Nieto N.C., Madigan J.E., Sykes J. (2008). Possible differential tropism in *Anaplasma phagocytophilum* strains in the western U.S.. Ann. N. Y. Acad. Sci..

[15] Margos G., Lane R.S., Fedorova N., Koloczek J., Piesman J., Hojgaard A., Sing A., Fingerle V. (2016). *Borrelia bissettiae* sp. nov. and *Borrelia californiensis* sp. nov. prevail in diverse enzootic transmission cycles. Int. J. Syst. Evol. Microbiol..

[16] Margos G., Vollmer S.A., Ogden N.H., Fish D. (2011). Population genetics, taxonomy, phylogeny and evolution of *Borrelia burgdorferi*
*sensu lato*. Infect. Genet. Evol..

[17] Schwan T.G., Schrumpf M.E., Karstens R.H., Clover J.R., Wong J., Daugherty M., Struthers M., Rosa P.A. (1993). Distribution and molecular analysis of Lyme disease spirochetes, *Borrelia burgdorferi*, isolated from ticks throughout California. J. Clin. Microbiol..

[18] Postic D., Assous M.V., Grimont P.A., Baranton G. (1994). Diversity of *Borrelia burgdorferi sensu lato* evidenced by restriction fragment length polymorphism of rrf (5S)-rrl (23S) intergenic spacer amplicons. Int. J. Syst. Evol. Microbiol..

[19] Lane R.S., Pascocello J.A. (1989). Antigenic characteristics of *Borrelia burgdorferi* isolates from ixodid ticks in California. J. Clin. Microbiol..

[20] Fedorova N., Kleinjan J.E., James D., Hui L.T., Peeters H., Lane R.S. (2014). Remarkable diversity of tick or mammalian-associated Borreliae in the metropolitan San Francisco Bay Area, California. Ticks Tick-Borne Dis..

[21] Donahue J.G., Piesman J., Spielman A. (1987). Reservoir competence of white-footed mice for Lyme disease spirochetes. Am. J. Trop. Med. Hyg..

[22] Massung R.F., Priestley R.A., Levin M.L. (2004). Transmission route efficacy and kinetics of *Anaplasma phagocytophilum* infection in white-footed mouse, *Peromyscus leucopus*. Vector-Borne Zoonotic Dis..

[23] Bockenstedt L.K., Wormser G.P. (2014). Review: Unraveling Lyme disease. Arthritis Rheumatol..

[24] Dumler J.S., Choi K.S., Garcia-Garcia J.C., Barat N.S., Scorpio D.G., Garyu J.W., Grab D.J., Bakken J.S. (2005). Human granulocytic anaplasmosis and *Anaplasma phagocytophilum*. Emerg. Infect. Dis..

[25] Keesing F., Hersh M.H., Tibbetts M., McHenry D.J., Duerr S., Brunner J., Killilea M., LoGiudice K., Schmidt K.A., Ostfeld R.S. (2012). Reservoir competence of vertebrate hosts for *Anaplasma phagocytophilum*. Emerg. Infect. Dis..

[26] Foley J.E., Kramer V.L., Weber D. (2002). Experimental ehrlichiosis in dusky footed woodrats (*Neotoma fuscipes*). J. Wildl. Dis..

[27] Eisen L., Eisen R.J., Mun J., Salkeld D.J., Lane R.S. (2009). Transmission cycles of *Borrelia burgdorferi* and *B. bissettii* in relation to habitat type in northwestern California. J. Vector Ecol..

[28] Salkeld D.J., Leonhard S., Girard Y.A., Hahn N., Mun J., Padgett K.A., Lane R.S. (2008). Identifying the reservoir hosts of the Lyme disease spirochete *Borrelia burgdorferi* in California: The role of the western gray squirrel (*Sciurus griseus*). Am. J. Trop. Med. Hyg..

[29] Lane R.S., Mun J., Eisen R.J., Eisen L. (2005). Western gray squirrel (Rodentia: Sciuridae): A primary reservoir host of *Borrelia burgdorferi* in Californian oak woodlands?. J. Med. Entomol..

[30] Nieto N.C., Foley J.E. (2009). Reservoir competence of the redwood chipmunk (*Tamias ochrogenys*) for *Anaplasma phagocytophilum*. Vector-Borne Zoonotic Dis..

[31] Swei A., Bowie V.C., Bowie R.C. (2015). Comparative genetic diversity of Lyme disease bacteria in northern Californian ticks and their vertebrate hosts. Ticks Tick-Borne Dis..

[32] Swei A., Ostfeld R.S., Lane R.S., Briggs C.J. (2011). Effects of an invasive forest pathogen on abundance of ticks and their vertebrate hosts in a California Lyme disease focus. Oecologia.

[33] Nieto N.C., Foley J.E. (2008). Evaluation of squirrels (Rodentia: Sciuridae) as ecologically significant hosts for *Anaplasma phagocytophilum* in California. J. Med. Entomol..

[34] Foley J., Stephenson N., Qurollo B., Breitschwerdt E. (2016). A putative marker for human pathogenic strains of *Anaplasma phagocytophilum* correlates with geography and host, but not human tropism. Ticks Tick-Borne Dis..

[35] Stephenson N., Hodzic E., Mapes S., Rejmanek D., Foley J. (2015). A real-time PCR assay for differentiating pathogenic *Anaplasma phagocytophilum* from an apathogenic, woodrat-adapted genospecies from North America. Ticks Tick-Borne Dis..

[36] Foley J., Hasty J., Lane R. (2016). Diversity of rickettsial pathogens in Columbian black-tailed deer and their associated keds (Diptera: Hippoboscidae) and ticks (Acari: Ixodidae). J. Vector Ecol..

[37] Foley J., Barlough J., Kimsey R., Madigan J., DeRock E., Poland A. (1998). *Ehrlichia* spp. in cervids from California. J. Wildl. Dis..

[38] Yabsley M.J., Davidson W.R., Stallknecht D.E., Varela A.S., Swift P.K., Devos J.C., Dubay S.A. (2005). Evidence of tick-borne organisms in mule deer (*Odocoileus hemionus*) from the western United States. Vector-Borne Zoonotic Dis..

[39] Wright S.A., Thompson M.A., Miller M.J., Knerl K.M., Elms S.L., Karpowicz J.C., Young J.F., Kramer V.L. (2000). Ecology of *Borrelia burgdorferi* in ticks (Acari: Ixodidae), rodents, and birds in the Sierra Nevada foothills, Placer County, California. J. Med. Entomol..

[40] Eisen L., Eisen R.J., Lane R.S. (2004). The roles of birds, lizards, and rodents as hosts for the western black-legged tick *Ixodes pacificus*. J. Vector Ecol..

[41] Newman E.A., Eisen L., Eisen R.J., Fedorova N., Hasty J.M., Vaughn C., Lane R.S. (2015). *Borrelia burgdorferi sensu lato* spirochetes in wild birds in northwestern California: Associations with ecological factors, bird behavior and tick infestation. PLoS ONE.

[42] Dingler R.J., Wright S.A., Donahue A., Madeco P., Foley J. (2014). Surveillance for *Ixodes pacificus* and the tick-borne pathogens *Anaplasma phagocytophilum* and *Borrelia burgdorferi* in birds from California’s Inner Coast Range. Ticks Tick-Borne Dis..

[43] Brown R.N., Peot M.A., Lane R.S. (2006). Sylvatic maintenance of *Borrelia burgdorferi* (Spirochaetales) in Northern California: Untangling the web of transmission. J. Med. Entomol..

[44] Salkeld D.J., Nieto N.C., Carbajales-Dale P., Carbajales-Dale M., Cinkovich S.S., Lambin E.F. (2015). Disease risk & landscape attributes of tick-borne *Borrelia* pathogens in the San Francisco Bay Area, California. PLoS ONE.

[45] Salkeld D.J., Cinkovich S., Nieto N.C. (2014). Tick-borne pathogens in northwestern California, USA. Emerg. Infect. Dis..

[46] Crowder C.D., Matthews H.E., Schutzer S., Rounds M.A., Luft B.J., Nolte O., Campbell S.R., Phillipson C.A., Li F., Sampath R. (2010). Genotypic variation and mixtures of Lyme *Borrelia* in *Ixodes* ticks from North America and Europe. PLoS ONE.

[47] Eisen R.J., Eisen L., Girard Y.A., Fedorova N., Mun J., Slikas B., Leonhard S., Kitron U., Lane R.S. (2010). A spatially-explicit model of acarological risk of exposure to *Borrelia burgdorferi*-infected *Ixodes pacificus* nymphs in northwestern California based on woodland type, temperature, and water vapor. Ticks Tick-Borne Dis..

[48] Lane R.S., Mun J., Parker J.M., White M. (2005). Columbian black-tailed deer (*Odocoileus hemionus columbianus*) as hosts for *Borrelia* spp. in northern California. J. Wildl. Dis..

[49] Eisen L., Eisen R.J., Chang C.C., Mun J., Lane R.S. (2004). Acarologic risk of exposure to *Borrelia burgdorferi* spirochaetes: Long-term evaluations in north-western California, with implications for Lyme borreliosis risk-assessment models. Med. Vet. Entomol..

[50] Eshoo M.W., Carolan H.E., Massire C., Chou D.M., Crowder C.D., Rounds M.A., Phillipson C.A., Schutzer S.E., Ecker D.J. (2015). Survey of *Ixodes pacificus* ticks in California reveals a diversity of microorganisms and a novel and widespread Anaplasmataceae species. PLoS ONE.

[51] Vredevoe L.K., Stevens J.R., Schneider B.S. (2004). Detection and characterization of *Borrelia bissettii* in rodents from the central California coast. J. Med. Entomol..

[52] Boyce W.M., Brown R.N., Zingg B.C., Lefebvre R.B., Lane R.S. (1992). First isolation of *Borrelia burgdorferi* in southern California. J. Med. Entomol..

[53] Sholty K.E. (2015). Strain Distribtution of *Borrelia burgdorferi* and *Anaplasma phagocytophilum* in Sciurids and Woodrats in Northwestern California. Master’s Thesis.

[54] Bissett M.L., Hill W. (1987). Characterization of *Borrelia burgdorferi* strains isolated from *Ixodes pacificus* ticks in California. J. Clin. Microbiol..

[55] Lane R.S., Fedorova N., Kleinjan J.E., Maxwell M. (2013). Eco-epidemiological factors contributing to the low risk of human exposure to ixodid tick-borne borreliae in southern California, USA. Ticks Tick-Borne Dis..

[56] Foley J., Ott-Conn C., Worth S.J., Poulsen A., Clifford D. (2014). An *Ixodes minor* and *Borrelia carolinensis* enzootic cycle involving a critically endangered Mojave Desert rodent. Ecol. Evol..

[57] Furman D.P., Loomis E.C. (1984). The Ticks of California (Acari: Ixodida).

[58] Bakken J.S., Dumler S. (2008). Human granulocytic anaplasmosis. Inf. Dis. Clin. N. Am..

[59] Madigan J. (1993). Equine ehrlichiosis. Vet. Clin. N. Am. Equine Pract..

[60] Carrade D.D., Foley J.E., Borjesson D.L., Sykes J.E. (2009). Canine granulocytic anaplasmosis: A review. J. Vet. Int. Sci..

[61] Hardalo C., Quagliarello V., Dumler J. (1995). Human granulocytic ehrlichiosis in Connecticut: Report of a fatal case. Clin. Inf. Dis..

[62] Walker D.H., Dumler J.S. (1996). Emergence of the ehrlichioses as human health problems. Emerg. Infect. Dis..

[63] Scorpio D.G., Dumler J.S., Barat N.C., Cook J.A., Barat C.E., Stillman B.A., DeBisceglie K.C., Beall M.J., Chandrashekar R. (2011). Comparative strain analysis of *Anaplasma phagocytophilum* infection and clinical outcomes in a canine model of granulocytic anaplasmosis. Vector-Borne Zoonotic Dis..

[64] Wormser G.P., Dattwyler R.J., Shapiro E.D., Halperin J.J., Steere A.C., Klempner M.S., Krause P.J., Bakken J.S., Strle F., Stanek G. (2006). The clinical assessment, treatment, and prevention of Lyme disease, human granulocytic anaplasmosis, and babesiosis: Clinical practice guidelines by the Infectious Diseases Society of America. Clin. Inf. Dis..

[65] Appel M.J., Allan S., Jacobson R.H., Lauderdale T.L., Chang Y.F., Shin S.J., Thomford J.W., Todhunter R.J., Summers B.A. (1993). Experimental Lyme disease in dogs produces arthritis and persistent infection. J. Infect. Dis..

[66] Greene C.E., Straubinger R., Levy S. (2012). Borreliosis. Infectious Diseases of the Dog and Cat.

[67] Hahn C., Mayhew I., Mackay R., Colahan P., Mayhew I., Merritt A., Moore J. (1999). Borreliosis. Equine Medicine and Surgery.

[68] Castro M.B., Wright S.A. (2007). Vertebrate hosts of *Ixodes pacificus* (Acari: Ixodidae) in California. J. Vector Ecol..

[69] Furman D., Catts E. (1982). Manual of Medical Entomology.

[70] Lane R.S., Brown R.N., Piesman J., Peavey C.A. (1994). Vector competence of *Ixodes pacificus* and *Dermacentor occidentalis* (Acari: Ixodidae) for various isolates of Lyme disease spirochetes. J. Med. Entomol..

[71] Gordus A.G. (1992). Prevalence of Lyme Borreliosis in Deer Mice and Ticks from Northeastern California. Ph.D. Thesis.

[72] Banerjee S. (1993). Isolation of *Borrelia burgdorferi* in British Columbia. Can. Commun. Dis. Rep..

[73] Banerjee S., Banerjee M., Smith J., Fernando K. (1994). Lyme disease in British Columbia—An update. B. C. Med. J..

[74] Damrow T., Freedman H., Lane R., Preston K. (1989). Is *Ixodes (Ixodiopsis) angustus* a vector of Lyme disease in Washington State?. West. J. Med..

[75] Peavey C.A., Lane R.S., Damrow T. (2000). Vector competence of *Ixodes angustus* (Acari: Ixodidae) for *Borrelia burgdorferi* sensu stricto. Exp. Appl. Acarol..

[76] Nieto N., Foley J., Bettaso J., Lane R. (2009). Reptile infection with *Anaplasma phagocytophilum*, the causative agent of granulocytic anaplasmosis. J. Parasitol..

[77] Lane R.S., Quistad G. (1998). Borreliacidal factor in the blood of the western fence lizard (*Sceloporus occidentalis*). J. Parasitol..

[78] Swei A., Ostfeld R.S., Lane R.S., Briggs C.J. (2011). Impact of the experimental removal of lizards on Lyme disease risk. Proc. R. Soc. Lond. B Biol. Sci..

[79] Majláthová V., Majláth I., Derdáková M., Víchová B., Pet’ko B. (2006). *Borrelia lusitaniae* and green lizards (*Lacerta viridis*), Karst Region, Slovakia. Emerg. Infect. Dis..

[80] Baldridge G.D., Scoles G.A., Burkhardt N.Y., Schloeder B., Kurtti T.J., Munderloh U.G. (2009). Transovarial transmission of *Francisella*-like endosymbionts and *Anaplasma phagocytophilum* variants in *Dermacentor albipictus* (Acari: Ixodidae). J. Med. Entomol..

[81] Westrom D., Anderson J. (1992). The distribution and seasonal abundance of deer keds (Diptera: Hippoboscidae) on Columbian black-tailed deer (*Odocoileus hemionus columbianus*) in northern California. Bull. Soc. Vector Ecol..

[82] Massung R.F., Mather T.N., Levin M.L. (2006). Reservoir competency of goats for the Ap-variant 1 strain of *Anaplasma phagocytophilum*. Infect. Immun..

[83] Massung R.F., Levin M.L., Munderloh U.G., Silverman D.J., Lynch M.J., Gaywee J.K., Kurtti T.J. (2007). Isolation and propagation of the Ap-Variant 1 strain of *Anaplasma phagocytophilum* in a tick cell line. J. Clin. Microbiol..

[84] Rudenko N., Golovchenko M., Grubhoffer L., Oliver J.H. (2011). *Borrelia carolinensis* sp. nov., a novel species of the *Borrelia burgdorferi* sensu lato complex isolated from rodents and a tick from the south-eastern USA. Int. J. Syst. Evol. Microbiol..

[85] Clark K.L., Oliver J.H., Grego J.M., James A.M., Durden L.A., Banks C.W. (2001). Host associations of ticks parasitizing rodents at *Borrelia burgdorferi* enzootic sites in south Carolina. J. Parasitol..

[86] Neumann L. (1902). Notes sur les Ixodides. Arch. Parasitol..

[87] Fairchild G. (1943). An annotated list of the bloodsucking insects, ticks and mites known from Panama. Am. J. Trop. Med..

[88] Poulsen A., Conroy C., Foley P., Ott-Conn C., Roy A., Brown R., Foley J. (2015). Ectoparasites of *Microtus californicus* and possible emergence of an exotic *Ixodes* species tick in California. J. Med. Entomol..

[89] Postic D., Garnier M., Baranton G. (2007). Multilocus sequence analysis of atypical *Borrelia burgdorferi sensu lato* isolates—Description of *Borrelia californiensis* sp. nov., and genomospecies 1 and 2. Int. J. Med. Microbiol..

[90] Scott J.M., Foley J. (2016). Detection of *Borrelia americana* in the avian coastal tick, *Ixodes auritulus* (Acari: Ixodidae), collected from a bird captured in Canada. Open J. Anim. Sci..

[91] Clark K.L., Leydet B., Hartman S. (2013). Lyme borreliosis in human patients in Florida and Georgia, USA. Int. J. Med. Sci..

[92] Strle F., Picken R.N., Cheng Y., Cimperman J., Maraspin V., Lotric-Furlan S., Ruzic-Sabljic E., Picken M.M. (1997). Clinical findings for patients with Lyme borreliosis caused by *Borrelia burgdorferi sensu lato* with genotypic and phenotypic similarities to strain 25015. Clin. Inf. Dis..

[93] Rudenko N., Golovchenko M., Ruzek D., Piskunova N., Mallatova N., Grubhoffer L. (2009). Molecular detection of *Borrelia bissettii* DNA in serum samples from patients in the Czech Republic with suspected borreliosis. FEMS Microbiol. Lett..

[94] Rudenko N., Golovchenko M., Mokráček A., Piskunová N., Růžek D., Mallatová N., Grubhoffer L. (2008). Detection of *Borrelia bissettii* in cardiac valve tissue of a patient with endocarditis and aortic valve stenosis in the Czech Republic. J. Clin. Microbiol..

[95] Fingerle V., Schulte-Spechtel U.C., Ruzic-Sabljic E., Leonhard S., Hofmann H., Weber K., Pfister K., Strle F., Wilske B. (2008). Epidemiological aspects and molecular characterization of *Borrelia burgdorferi* sl from southern Germany with special respect to the new species *Borrelia spielmanii* sp. nov.. Int. J. Med. Microbiol..

[96] Girard Y.A., Fedorova N., Lane R.S. (2011). Genetic diversity of *Borrelia burgdorferi* and detection of *B. bissettii*-like DNA in serum of north-coastal California residents. J. Clin. Microbiol..

[97] Zeidner N.S., Burkot T.R., Massung R., Nicholson W.L., Dolan M.C., Rutherford J.S., Biggerstaff B.J., Maupin G.O. (2000). Transmission of the agent of human granulocytic ehrlichiosis by *Ixodes spinipalpis* ticks: Evidence of an enzootic cycle of dual infection with *Borrelia burgdorferi* in northern Colorado. J. Infect. Dis..

[98] Linsdale J.M., Tevis L.P. (1951). The Dusky-Footed Wood Rat; A Record of Observations Made on the Hastings Natural History Reservation.

[99] Durand J., Jacquet M., Paillard L., Rais O., Gern L., Voordouw M.J. (2015). Cross-immunity and community structure of a multiple-strain pathogen in the tick vector. Appl. Environ. Micrbiol..

[100] Foley J., Rejmanek D., Foley C.W., Matocq M. (2016). Fine-scale genetic structure of woodrat populations (Genus: *Neotoma*) and the spatial distribution of their tick-borne pathogens. Ticks Tick-Borne Dis..

[101] Fleer K.A., Foley P., Calder L., Foley J.E. (2011). Arthropod vectors and vector-borne bacterial pathogens in Yosemite National Park. J. Med. Entomol..

[102] Lane R.S., Mun J., Stubbs H. (2009). Horizontal and vertical movements of host-seeking *Ixodes pacificus* (Acari: Ixodidae) nymphs in a hardwood forest. J. Vector Ecol..

[103] Foley J., Piovia-Scott J. (2014). Vector biodiversity did not associate with tick-borne pathogen prevalence in small mammal communities in northern and central California. Ticks Tick-Borne Dis..

[104] Stuen S., Granquist E.G., Silaghi C. (2013). *Anaplasma phagocytophilum*—A widespread multi-host pathogen with highly adaptive strategies. Front. Cell. Inf. Microbiol..

[105] Nieto N.C., Foley J.E. (2009). Meta-Analysis of coinfection and coexposure with *Borrelia burgdorferi* and *Anaplasma*
*phagocytophilum* in humans, domestic animals, wildlife, and *Ixodes ricinus*-complex ticks. Vector-Borne Zoonotic Dis..

[106] Jahfari S., Coipan E.C., Fonville M., Van Leeuwen A.D., Hengeveld P., Heylen D., Heyman P., Van Maanen C., Butler C.M., Földvári G. (2014). Circulation of four *Anaplasma phagocytophilum* ecotypes in Europe. Parasites Vectors.

[107] Bown K., Begon M., Bennett M., Woldehiwet Z., Ogden N. (2003). Seasonal dynamics of *Anaplasma phagocytophila* in a rodent-tick (*Ixodes trianguliceps*) system, United Kingdom. Emerg. Infect. Dis..

[108] Baráková I., Derdáková M., Carpi G., Rosso F., Collini M., Tagliapietra V., Ramponi C., Hauffe H.C., Rizzoli A. (2014). Genetic and ecologic variability among *Anaplasma phagocytophilum* strains, northern Italy. Emerg. Infect. Dis..

[109] Blaňarová L., Stanko M., Carpi G., Miklisová D., Víchová B., Mošanský L., Bona M., Derdáková M. (2014). Distinct *Anaplasma phagocytophilum* genotypes associated with *Ixodes trianguliceps* ticks and rodents in central Europe. Ticks Tick-Borne Dis..

[110] Rar V.A., Epikhina T.I., Yakimenko V.V., Malkova M.G., Tancev A.K., Bondarenko E.I., Ivanov M.K., Tikunova N.V. (2014). Genetic variability of *Anaplasma phagocytophilum* in ticks and voles from *Ixodes persulcatus/Ixodes trianguliceps* sympatric areas from western Siberia, Russia. Ticks Tick-Borne Dis..

